# Fosfomycin Pharmacokinetic Profile in Plasma and Urine and Quantitative Estimation in Prostate and Seminal Vesicles after One and Two Consecutive Doses of Oral Fosfomycin Trometamol in Healthy Male Volunteers

**DOI:** 10.3390/antibiotics11111458

**Published:** 2022-10-22

**Authors:** Andrea F. D. Di Stefano, Milko M. Radicioni, Francesca Morano, Alessandra Gentili, Elena Mallat, Dario Cuomo, Tonia Mazzarella, Veronica Di Fonzo

**Affiliations:** 1 CROSS Research S.A., Via F. A. Giorgioli, 14, CH-6864 Arzo, Switzerland; 2 Ardena Bioanalysis B.V., W. A. Scholtenstraat 7, NL-9403 AJ Assen, The Netherlands; 3 Zambon S.p.A., Global Medical Affairs, Via Lillo del Duca 10, I-20091 Bresso, Italy; 4 Zambon S.p.A., Regulatory Affairs, Via Lillo del Duca 10, I-20091 Bresso, Italy

**Keywords:** fosfomycin trometamol, pharmacokinetics, male genital tract, prostate, noninvasive, quantitation

## Abstract

The present Phase I study investigated, for the first time, fosfomycin pharmacokinetics in humans after two 3 g doses of fosfomycin trometamol administered 27 h apart, according to the dose regimen recommended for the prophylactic indication for transrectal prostate biopsy in adult men. Plasma, urine and seminal plasma concentrations were measured after one and two consecutive doses in 24 healthy men, representative of the target population of the prophylactic indication. Prostate and seminal vesicle concentrations were estimated based on seminal plasma concentrations using a one-step regression method. The exposure to fosfomycin was very similar in rate (C_max_, t_max_) after one and two doses. The AUC showed a minimal increment. On average, the apparent volume of distribution was high (>100 L), and the mean clearance had an intermediate value. The total amount and dose fraction of fosfomycin excreted in urine showed a small increment after two doses. The renal clearance was about 5 L/h. The fosfomycin concentration in the prostate and seminal vesicles showed that the antibiotic increased on average after two consecutive doses. This result confirmed the ability of fosfomycin to distribute into the prostate and into seminal vesicles after one single dose and that a two consecutive dose regimen increases the antibiotic availability inside these peripheral tissues.

## 1. Introduction

Fosfomycin is a bactericidal broad-spectrum antibiotic first isolated in 1969 from cultures of *Streptomyces fradiae* and *Pseudomonas syringae* [1,2]. Fosfomycin, which is currently produced using a synthetic process, is a low-molecular weight (138 g/mol), highly polar phosphonic acid derivative (*cis*–1,2-epoxypropyl phosphonic acid) that represents its own class of antibiotics [3].

The drug acts by inhibiting the formation of peptidoglycan, which results in bacterial lysis. Both Gram-positive and Gram-negative bacteria require peptidoglycan synthesis, which means that fosfomycin has a very broad spectrum of action and is active against the main genera in clinical practice.

Many bacterial species can develop various forms of resistance to fosfomycin, including changes or mutations in the biosynthesis pathway of the target molecule of the antibiotic, decreased fosfomycin uptake and fosfomycin inactivation reactions [2,4]. However, despite more than 30 years of use, there are low rates of resistance to fosfomycin which has preserved its high activity against multidrug and extensively drug resistant isolates, including extended-spectrum β lactamase-producing *Escherichia coli* strains [5,6].

The use of various available formulations of the antibiotic (as disodium salt for the intravenous route and as calcium or trometamol salt for the oral route) significantly increased due to a considerable rise in incidences of multidrug-resistant micro-organisms against which fosfomycin constitutes, alone or in combination, an alternative treatment option [4,7]. Consequently, the European Medicines Agency (EMA) has started reviewing accumulated information on the use of fosfomycin and the information from new clinical trials.

In recent years, clinical research in the field of urological manoeuvres has focused on antimicrobial prophylaxis for subjects undergoing transrectal prostate biopsy, as it is one of most frequently performed urological procedures and the common practice to obtain a histological diagnosis of prostatic carcinoma. Post-transrectal prostate biopsy infections are mostly caused by *Enterobacteriaceae*, particularly *E. coli*, which is the predominant aerobic member of the human bowel flora. Crucially, *E. coli* has evolved to become more frequently antibiotic-resistant over the past two decades, including a resistance developed against fluoroquinolones which were the commonly used chemoprophylactic agents in transrectal prostate biopsy thanks to a good penetration into the prostate [8,9]. In perioperative chemoprophylaxis, a sufficiently high antibiotic concentration is essential from the beginning of surgery, whether it is a biopsy or a surgical excision of an organ. Effective antibiotic concentrations guarantee an antimicrobial effect and hinder the bacterial colonization of the operative field to prevent wound infection and possible sepsis. An optimal tissue distribution and half-life of the antibiotics would also exceed the critical period of postoperative infection, which usually lasts until a few hours after the surgical intervention [10,11]. With the aim of proving the above-mentioned kinetic properties with respect to fosfomycin, the present Phase I study was designed to investigate, for the first time, the fosfomycin pharmacokinetic profile after two doses of fosfomycin trometamol administered according to the currently approved dose regimen, namely, “one 3 g fosfomycin trometamol sachet 3 h before surgery and a second dose of 3 g to be given 24 h after surgery” recommended for the indication “Perioperative antibiotic prophylaxis for transrectal prostate biopsy in adult men”, thus satisfying a request by the EMA Committee for Medicinal Products for Human Use.

## 2. Materials and Methods

### 2.1. Ethical Procedures

The study was approved by the independent ethics committee of Canton Ticino on 08FEB21 with reference number EC project ID: 2020-03039, CE 3796. The Swiss Federal Health Authorities (Swissmedic) authorised the study on 11MAR21 and assigned the reference number 2021DR1041. Subjects did not undergo any study procedure before signing the written informed consent form. The study was performed at the Phase I Unit of CROSS Research S.A., Arzo, Switzerland, in compliance with the Swiss ordinance on clinical trials in human research and in accordance with the Declaration of Helsinki and GCP. The first subject was enrolled on 18MAR21, and the last subject completed the trial on 12APR21.

### 2.2. Study Design

The present study was a single and multiple dose, single-centre, open-label, one-way, pharmacokinetics, safety and tolerability Phase I clinical study. Primary objective was to evaluate fosfomycin pharmacokinetics in plasma and urine in healthy men after one and two consecutive doses of fosfomycin trometamol. Secondary objectives of the study were to estimate fosfomycin concentrations in prostate and seminal vesicles in healthy men and to evaluate safety and tolerability of the treatment.

The selected study population were healthy men who were deemed to be representative of the target population of the fosfomycin prophylactic indication, i.e., “Perioperative antibiotic prophylaxis for transrectal prostate biopsy”. In fact, men undergoing transrectal prostate biopsy are otherwise healthy asides from the suspicion of prostate carcinoma that has prompted the procedure. Therefore, there is no reason to suppose that the target population of the fosfomycin prophylactic indication should exhibit different fosfomycin pharmacokinetics to healthy men.

The antibiotic concentrations in the prostatic tissue were not directly measured in the male accessory gland, considering that tissue biopsies are an invasive procedure and not susceptible to repeated sampling within a dosing interval. Instead, a fractioned ejaculate sampling was selected to estimate fosfomycin concentrations in the seminal vesicles and prostate applying a method previously described by Hendrix CW et al. [12,13,14,15]. Knowing the sequential release into the urethra of fluid from different male accessory glands and incomplete mixing of these fluids prior to emergence from the urethra, the relative contribution from each gland was quantifiable using measurement of biochemical markers: fructose for the seminal vesicle and prostate-specific antigen (PSA) for the prostate [13,14,15]. Once the glandular contribution to each fraction was determined, fosfomycin concentration measured in each fraction allowed calculation of its concentration in each of the glands. The procedure could be repeated twice. In fact, the split ejaculate was collected on Day 1 after the single dose and on Day 4 after two consecutive doses of fosfomycin trometamol. As a collection time point, 2.5 h postdose was selected, at which time the peak plasma concentration of fosfomycin was expected to be attained [16,17,18,19]. 

### 2.3. Study Population and Criteria for Inclusion

Healthy men were included into the trial according to the following main inclusion criteria: (i) aged from 40 to 70 years, (ii) good health based on medical history, physical examination, a 12-lead electrocardiogram (ECG), routine haematology, blood chemistry and urine tests and (iii) willingness to provide written informed consent. 

Main exclusion criteria were usual criteria for pharmacokinetics studies, namely, (i) intake of any medication, (ii) a history of drug, caffeine (>5 cups coffee/tea/day) or tobacco (≥10 cigarettes/day) abuse, (iii) history of alcohol consumption in excess of two drinks per day, as defined by the U.S.D.A. dietary guidelines and (iv) recent participation in clinical studies of other experimental drugs. 

The study was descriptive. Therefore, the sample size was not derived from a statistical power calculation. A total of 24 subjects were planned to be enrolled in the study. 

The subjects were confined from the evening preceding the first administration (study Day −1) until the evening of Day 5. 

### 2.4. Investigational Treatments and Dose Regimen

All the subjects enrolled in the study received the same treatment. On Day 1, they received one dose of 3 g of fosfomycin (as fosfomycin trometamol) under fasting conditions at 07:00 ± 1 h. After a wash-out of exactly 48 h, the subjects received one dose of 3 g under fasting conditions at 07:00 ± 1 h on Day 3, followed by a second 3 g dose exactly 27 h later, on Day 4, at 10:00 ± 1 h.

No medication, including over the counter and herbal remedies, was allowed for two weeks before the start of the study and during the whole study duration. Notably, metoclopramide, domperidone and other prokinetic agents were forbidden. Paracetamol was allowed as therapeutic countermeasure against adverse events according to the opinion of the investigator. 

The subjects were recommended to refrain from ejaculation starting from 48 h before the first study dose and during the whole duration of the study except for the two fractioned semen collections scheduled on Days 1 and 4. 

During confinement, the subjects did not consume any food or drinks (except water) for at least 10 h (i.e., overnight) before the single and the last multiple dose. On Day −1, a standardized light dinner was served, and then the subjects remained fasted until 5 h postdose. On Day 3, a standardized light dinner was served, as on Day −1, and then the subjects remained fasted until 3 h after dosing, which was at 10:00 ± 1 h on Day 4. All other meals served during the confinement were standardized. One cup of coffee or tea was allowed after each meal only; any other coffee, tea or food containing xanthines (i.e., coke, chocolate, etc.), alcohol and grapefruit were forbidden during confinement. Of note, grapefruit and alcohol were forbidden from 24 h before the first study dosing until the end of the study.

The subjects were allowed to smoke one cigarette after each meal during confinement.

During confinement, routine ambulant daily activities were strongly recommended, but hazardous, strenuous or athletic activities were not permitted.

### 2.5. Pharmacokinetics Variables and Data Analysis

Both after one dose (Day 1) and after two doses (Day 4), the following parameters were measured and/or calculated for plasma and urine fosfomycin according to a noncompartmental model using the validated software WinNonLin^®^ 6.3 (Pharsight Corporation, Princeton, NJ, USA): C_max_, t_max_, λ_z_ (calculated, if feasible, using log-linear regression using at least 3 points), t_1/2_ (calculated as ln2/λ_z_), AUC_0–t_ (area under the concentration–time curve from the dose to the last observed concentration time t, calculated using the linear trapezoidal method), AUC_0–__∞_, AUC_0–12_, AUC_0–24_, V_z_ (apparent volume of distribution associated with the terminal slope following extravascular administration divided by the fraction of dose absorbed, calculated, if feasible, as dose/(AUC_0–__∞_ × λ_z_)), Cl_t_ (calculated, if feasible, as dose/AUC_0–__∞_), Ae_0–t_ (defined as total amount of fosfomycin excreted in urine from dosing up to 36 h), Fe_0–t_ (defined as total fraction of fosfomycin dose excreted in urine from dosing up to 36 h) and Cl_r_ (calculated, if feasible, as Ae_0–t_/AUC_0–__∞_).

The following parameters were measured and/or calculated using the same software, after the 2nd multiple dose (Day 4): C_max,ss_, t_max,ss_, C_min,ss_, AUC_0–t,ss_, AUC_τ,ss_, C_ave,ss_ and PTF% (calculated as (C_max,ss_ − C_min,ss_)/C_ave,ss_ × 100).

### 2.6. Estimation of Fosfomycin Concentration in Prostate and Seminal Vesicles

The following variables were defined: *A* as concentration of PSA in prostatic secretions (calculated), *a_j_* as concentration of PSA in fraction j of seminal plasma (measured), *B* as concentration of fructose in seminal vesicle secretions (calculated), *b_j_* as concentration of fructose in fraction j of seminal plasma (measured), *C_p_* as concentration of fosfomycin in prostate (calculated), *C_v_* as concentration of fosfomycin in seminal vesicle (calculated) and *c_j_* as concentration of fosfomycin in the fraction j of seminal plasma (measured). 

**Figure 1:**
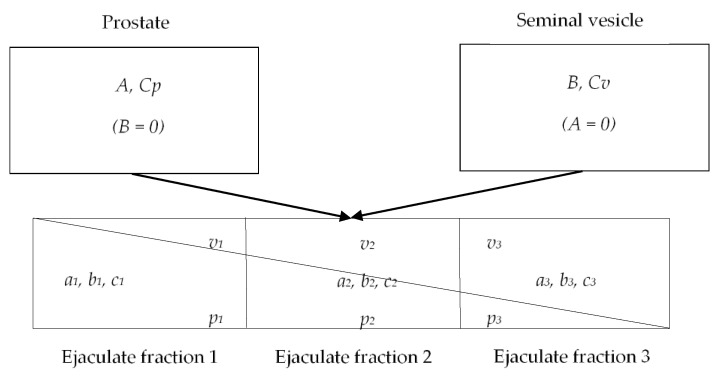
Schematic representation of split-ejaculate calculations based on prostate and seminal vesicle contributions to semen. *A*: concentration of PSA in prostatic secretions (calculated), *a_j_*: concentration of PSA in fraction *j* of seminal plasma (measured), *B*: concentration of fructose in seminal vesicle secretions (calculated), *b_j_*: concentration of fructose in fraction *j* of seminal plasma (measured), *v_j_*: proportion of seminal vesicle secretion in fraction *j* of seminal plasma (calculated), *C_P_*: concentration of fosfomycin in prostate (calculated), *C_V_*: concentration of fosfomycin in seminal vesicles (calculated) and *c_j_*: concentration of fosfomycin in the fraction *j* of seminal plasma (measured).

C*_P_* and C*_V_* were estimated using SAS^®^ version 9.3 (TS1M1) based on fosfomycin, fructose and PSA concentrations measured in seminal plasma, *c_s_*, *b* and *a*, respectively, as previously described [10,11,12]. The methodology postulates the possibility to collect the very first, infinitesimal, unmixed semen fraction, which would show PSA and fructose concentration as follows: *a_j_* = *A* and *b_j_* = 0, with PSA at its concentration inside the prostate, A and fructose still absent. Similarly, in the last fraction, *a_j_* = 0 and *b_j_* = *B*. Given that the real concentrations are a result of mixing within the urethra, the above-mentioned conditions are only postulated. However, the intermediate fractions represent a progression between these 2 extreme conditions as shown in Figure 1. The profile of PSA vs. fructose is therefore linear with a negative slope, and the axis intercepts indicate concentrations in cognate glands of origin.

Applying the one-step regression method for the estimation, as previously published by Ndovi T. et al. [15] and using the gradients derived from PSA and fructose seminal plasma concentrations in the same ejaculate fractions (*b* and *a*), *c_s_* allowed the estimate of *C_P_* and *C_V_*.

### 2.7. Sample Collection, Handling and Analytics 

Plasma concentrations of fosfomycin were measured at the following times on Days 1–2 after the single dose and on Days 4–5 after two consecutive doses:

- predose (0), 0.5, 1, 1.5, 2, 2.5, 3, 3.5, 4, 6, 8, 10, 12, 16, 24, 30 and 36 h postdose;

Urine was collected during the following time intervals on Days 1–2 after the single dose and on Days 4–5 after two consecutive doses:

- 0–4, 4–8, 8–12, 12–16, 16–24 and 24–36 h postdose.

Blood samples for pharmacokinetic analysis were collected using an indwelling catheter with switch valve and transferred from the catheter with a syringe into heparinized tubes (Li-heparin). The samples were stored on ice for a maximum of 20 min and then centrifuged at 4 °C for 10 min at 2500× *g* to obtain plasma. Each plasma sample was immediately divided into 3 aliquots in prelabelled polypropylene tubes. 

During each urine collection interval, urine was collected into containers and kept refrigerated at approximately 4 °C. Before the end of each collection interval, bladder was emptied, volume was measured and, after thorough mixing, 3 aliquots were prepared in polypropylene tubes.

For ejaculate collection, the subjects were instructed to masturbate and ejaculate their semen at about 2.5 h postdose on Day 1 after the single dose and on Day 4 after the second consecutive dose and, in any case, before the 2.5 h postdose blood sampling. The subjects were instructed to collect their semen into a 5-compartment plastic collection tray in a single pass across the long axis of the tray as described in the literature [11]. The subjects were instructed to collect semen in at least 3 (preferably 5) of the tray compartments, which represented 3 discrete fractions (“split ejaculate”). The subjects were instructed to wipe away, whenever possible, visible pre-ejaculate fluid that appeared at the urethral orifice prior to ejaculation. After collection, the tray of each individual subject was covered with its hinged lid and kept for 30 min at room temperature to allow the semen to liquefy. The liquefied semen, separately from each of the 5 compartments, was transferred using positive displacement pipettes into distinct tared prelabelled sterile conical centrifuge tubes. The weight of each fraction of split ejaculate was measured and recorded. The samples were centrifuged at 800× *g* for 10 min at 4 °C.

After centrifugation, supernatant seminal plasma of each tube was collected into one prelabelled polypropylene tube, thus obtaining 5 aliquots, i.e., one for each tray compartment. Each aliquot was weighed, and weights recorded. All the biological samples were stored frozen at ≤−70 °C at the Phase I Unit. 

### 2.8. Bioanalysis

The concentration of fosfomycin in plasma, urine and seminal plasma was determined at Ardena Bioanalysis BV, The Netherlands, using fully validated LC-MS/MS methods with a lower quantification limit (LQL) of 0.500 µg/mL in plasma, 0.0500 mg/mL in urine and 0.500 µg/mL in seminal plasma. The concentration of fructose and PSA in seminal plasma was determined at the same bioanalytical laboratory using, for the former, a qualified LC-MS/MS method and a qualified ELISA, for the latter.

Fosfomycin and its internal standard were extracted by protein precipitation from plasma and seminal plasma samples and by simple dilution from urine samples. Subsequently, the extracts were injected into the chromatographic system using an Acquity BEH Amide HILIC column with 1.7 µm particle size and with the dimensions 50 mm × 2.1 mm. A mobile phase consisting of a solution of 20 mM NH_4_HCO_3_ at pH 10 and acetonitrile was used for separation. The detection was performed using an API 4000™ LC-MS/MS SCIEX System equipped with an electrospray ionization (ESI) ion source operating in the negative multiple reaction monitoring mode.

The LC-MS/MS method for the determination of fosfomycin in plasma, urine and seminal plasma produced accurate and precise results. With respect to the accuracy of the method, the validation study revealed between-run biases for the quality control (QC) samples at the levels LLOQ (LQC), low (QC-Low), medium (QC-Medium), high (QC-High) of: −0.9, 0.6, 0.5 and 0.3% in plasma; −2.6, −0.1, 1.6 and −2.1% in urine; and −1.9, −3.3, −5.5 and −5.6% in seminal plasma, respectively. The precision results (expressed as total CV%) were as follows: 10.3, 5.0, 4.2 and 4.9% in plasma; 3.6, 3.3, 1.9 and 1.5% in urine; and 9.0, 3.8, 4.5 and 4.8% in seminal plasma for the LQC, QC-Low, QC-Medium and QC-High samples, respectively. 

Intra-run CVs (repeatability) were: 9.7, 5.7, 6.5 and 4.0% in plasma; 3.4, 1.9, 1.0 and 2.1% in urine; and 7.1, 3.7, 3.8 and 2.7% in seminal plasma for the LQC, QC-Low, QC-Medium and QC-High samples, respectively. The calibration range covered 0.500–100 µg/mL in plasma and seminal plasma, and 0.0500–10.0 mg/mL in urine. 

The long-term stability of fosfomycin in plasma and seminal plasma samples was assessed for a period of 93 days and 94 days, respectively, at ≤−18 °C and ≤−70 °C and was found valid. In parallel, the long-term stability of fosfomycin in urine samples was assessed for a period of 96 days at ≤−18 °C and ≤−70 °C and was found valid.

Fructose and its internal standard were extracted from seminal plasma by protein precipitation. The analytes were separated in an XBridge BEH Amide chromatographic column with 2.5 µm particle size and with the dimensions 100 mm × 3.00 mm. A mobile phase consisting of a solution of 10 mM NH_4_HCO_3_, 1% NH_4_ and acetonitrile was used for separation. The detection was performed using an API 4000™ LC-MS/MS SCIEX System equipped with an electrospray ionization (ESI) ion source operating in the negative multiple reaction monitoring mode. The qualification of the determination of fructose in seminal plasma revealed within-run biases for the quality control (QC) samples at the levels LLOQ (LQC), low (QC-Low), medium (QC-Medium), high (QC-High) of −12.5, −0.2, −3.6, −3.2 and −6.4%, respectively. The calibration range covered 100–10,000 µg/mL in seminal plasma. Bench top stability of 20 h at room temperature and two freeze-thaw cycles at the temperature ≤−70 °C were also demonstrated.

PSA in seminal plasma was analysed using a quantitative sandwich ELISA; specifically, the Human Kallikrein 3/PSA Quantikine ELISA Kit of R&D Systems (Minneapolis, MN, USA) was used for analysis. The qualified method is suitable for the measurement of PSA in seminal plasma in the concentration range 0.938–60.0 ng/mL, providing selective, precise and reproducible measurements. Bench top stability of 18 h at room temperature and four freeze-thaw cycles at ≤−70 °C were also demonstrated.

Plasma, seminal plasma and urine samples were stored at ≤−70 °C at the laboratory facilities before and after analysis.

### 2.9. Safety Variables

The safety variables included the recording of adverse events during the whole study duration, blood pressure, heart rate and body weight measurements at screening, predose, postdose and upon discharge, physical examinations, ECG and routine blood chemistry, haematology and urinalysis laboratory assays performed at screening and upon final discharge.

## 3. Results

### 3.1. Disposition of subjects

Twenty-four healthy men aged 40 to 63 years were enrolled as planned. The baseline demographic data are summarised in Table 1.

After inclusion, all 24 subjects received the complete study treatment, and all of them completed the study as per the protocol. The pharmacokinetics analysis of plasma and urine fosfomycin on Days 1 and 4 included all 24 subjects, while 22 out of the 24 enrolled subjects were included in the analysis of fosfomycin in the seminal plasma and the estimation of prostatic and seminal vesicle fosfomycin due to missing ejaculate collections either on Day 1 or on Day 4.

### 3.2. Fosfomycin Plasma Levels

**Figure 2:**
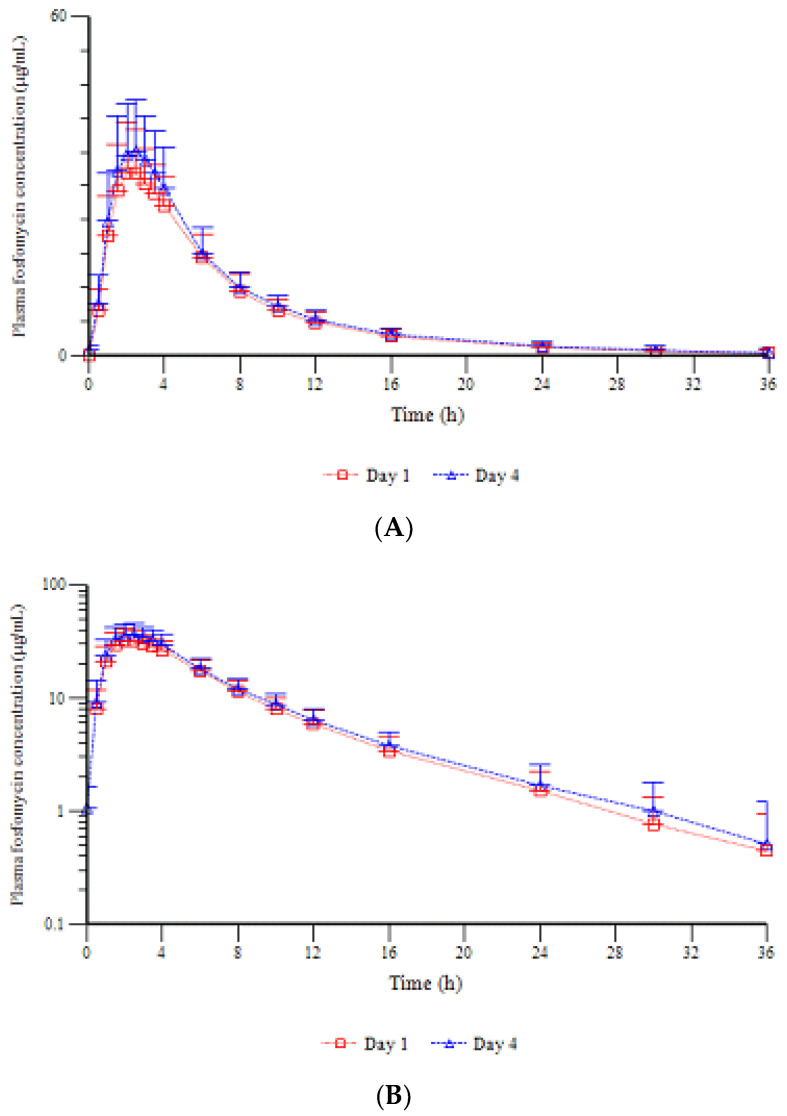
Mean (+SD) plasma fosfomycin (µg/mL) vs. time profiles after single and two consecutive doses of fosfomycin trometamol. Linear scale (**A**) and semilogarithmic scale (**B**).

Figure 2 shows the mean fosfomycin plasma levels vs. time profiles measured on Day 1, up to 36 h after the single dose and on Day 4, up to 36 h after two consecutive doses of fosfomycin trometamol. 

On Day 1, the fosfomycin levels rapidly increased in the plasma after the single dose. The highest observed concentration was 32.271 ± 7.634 µg/mL, attained on average 2.5 h postdose. After the peak, fosfomycin decreased in the plasma until 36 h postdose, when it was below the quantification limit in half of the subjects. 

On Day 4, predose fosfomycin concentrations were on average quantifiable (mean ± SD: 1.073 ± 0.558 µg/mL) as after the single dose, the highest observed concentration was attained 2.5 h postdose and amounted to 36.196 ± 9.191 µg/mL. After the peak, the antibiotic concentrations declined in the plasma, showing a superimposable curve. 

The elimination phase could be clearly defined for all the subjects after one and two doses. This means that the criteria for extrapolation to infinity established in the protocol were met for all the subjects and that the parameters λ_z_, t_1/2_, AUC_0–__∞_, AUC_extra_, V_z_ and Cl_t_ could be calculated. The main pharmacokinetic parameters of plasma fosfomycin on Days 1 and 4 are presented in Table 2, with the steady-state parameters of plasma fosfomycin on Day 4.

### 3.3. Fosfomycin in Urine

Consistent with the plasma concentration profile, the highest partial excretion of fosfomycin after both one and two consecutive doses occurred in the first two collection intervals 0–4 and 4–8 h postdose. In all of the following collection intervals, the fosfomycin excretion decreased on average after both one and two doses. 

The main pharmacokinetic parameters of urine fosfomycin on Days 1 and 4 are presented in Table 3.

### 3.4. Estimated Fosfomycin Concentrations in Prostate and Seminal Vesicles

The mean fosfomycin concentrations measured in the seminal plasma on Days 1 and 4 are reported in Table 4. 

Both on Day 1 and Day 4, fosfomycin was quantifiable in the majority of the ejaculate fractions from the majority of subjects, although some ejaculate fractions were missing for a few subjects, or some aliquot had an insufficient volume for the bioanalysis of all three analytes. 

The estimated fosfomycin concentrations in the prostate and seminal vesicles on Days 1 and 4 are presented in Table 5.

*C_P_* and *C_V_* clearly showed that the antibiotic concentration in both the peripheral tissues increased on average (both in mean and 95% CI) after two consecutive doses compared to the single dosing. While *C_P_* and *C_V_* increased on average from 9.98 to 34.42 µg/mL and from 2.08 to 14.95 µg/mL, respectively, the increment in the plasma concentration at the corresponding time point of 2.5 h postdose was from 32.271 ± 7.634 µg/mL to 36.196 ± 9.191 µg/mL. 

### 3.5. Safety

Altogether, 14 treatment emergent adverse events occurred to five subjects at a frequency of 20.8%, as shown in Table 6. The outcome of all AEs was resolution. All but one of the events were judged as related to the study treatment. 

The most frequent event, both among the overall and the treatment-related ones, was diarrhoea (verbatim “loose stool”). Occurrences were generally single events and intermittent for some subjects. Most occurrences were mild in severity except for two occurrences which led the affected subjects to not complete the urine collection as planned in the 8–12 h postdose interval. All the reported episodes spontaneously resolved. No subject was excluded from the analysis due to the occurrence of loose stools. All reported episodes occurred hours after the attainment of the concentration peak in the plasma. Considering both the individual plasma fosfomycin concentration vs. the time profiles and the time of the onset of the reported episodes, any impact of loose stools on the fosfomycin kinetic profile in the concerned subjects was excluded. 

No significant effects of the study treatment on blood pressure, heart rate, body weight, ECG or laboratory parameters were observed.

## 4. Discussion

Fosfomycin pharmacokinetics was investigated in the plasma and urine up to 36 h postdose after one and then after two consecutive doses in 24 healthy men, representative of the target population of the fosfomycin trometamol prophylactic indication. The fosfomycin kinetic profile (rates of absorption and elimination) after two consecutive doses of oral fosfomycin trometamol was investigated for the first time in this study. The study results show that both the observed plasma fosfomycin absorption/distribution curve part and the elimination curve part as well as the extent of the concentration peak were almost superimposable after one single or two consecutive doses. Slight increments were only observed in the rate and extent of exposure, which were devoid of significance, considering their standard deviations. On average, the peak concentration was reached in a median time of 2.5 h after one dose and of 2.0 h after two consecutive doses, and the Day 4 C_max_ was, on average, only slightly higher than after a single dose. The AUC_0–t_, AUC_0–12_, AUC_0–24_, AUC_0–t,ss_ and AUC_τ,ss_ showed just a minimal increment. All these results together with a high PTF% indicated that fosfomycin did not reach steady-state conditions after only two consecutive doses, which is consistent with the early literature about fosfomycin pharmacokinetics reporting that the steady-state was attained by the antibiotic after three or four doses [20]. These data are also consistent with a more recent study which did not show any steady-state conditions of fosfomycin in the blood stream of 18 healthy subjects at Day 5 after they received 3 g doses of fosfomycin trometamol daily for 5 days and on Days 1, 3 and 5, according to a randomized cross-over design [21]. 

On average, the apparent volume of distribution V_z_ was high. The values >100 L indicated that fosfomycin can also reach deep tissue compartments, including fatty tissues. The fosfomycin clearance had an intermediate value, slightly higher than the mean glomerular filtration rate (>9 L/h). In conclusion, half-life, apparent volume of distribution and clearance observed in the present study indicate that fosfomycin is widely distributed throughout the tissues and then eliminated at an intermediate rate. 

The total amount of fosfomycin excreted in urine (Ae_0–t_) showed a difference between Day 1 and 4, though amounting to a small increment after two consecutive doses compared to the single dose, in agreement with the observations performed in the plasma. The observed small increments, as for the plasma parameters, were devoid of any significance, as shown by their standard deviations. The total fraction of the administered 3 g dose, excreted in urine as unchanged fosfomycin up to 36 h (Fe_0–t_), was on average similar between the single and two consecutive doses and represented nearly half of the administered dose. Furthermore, renal clearance was approximately 5 L/h after both the single and two consecutive doses.

The mean C_max_ values observed in the present study after a single dose were slightly higher than those observed in previous studies of fosfomycin trometamol, whose values ranged between 22–32 µg/mL [2,16,18,21,22,23,24,25], the highest of which (32 µg/mL [16]) was slightly lower than the C_max_ reported for the single dose in the present study (35.30 ± 7.86 µg/mL). Notably, the literature median t_max_ (ranging 2.0–2.5 h [2,16,18,21,22,23,24,25]), percentage excretion (Fe_0–t_) (ranging 32–50% [2,16,18,21,22,23,24,26]) and AUCs (ranging 181–228 µg/mL∙h [2,16,18,23,25]) were generally consistent with the present study data. 

The mean t_1/2_ values were on average higher than all half-life values calculated previously in older studies (ranging 2.4–5.6 h [2,16,18,21,22,23,24,26]) with the only exception of the value reported by Muller-Serieys et al., indicating a mean half-life of 7.31 ± 1.74 h [25], nearer to the present study result. 

On average, the apparent volume of distribution V_z_—higher than 100 L and therefore higher than the total body fluid volume—was consistent with the value reported in the summary product characteristics [19] and previously by Wenzler et al. [21] (139–172 L), Muller-Serieys et al. [25] (145 L) and Borsa et al. [26] (2.4 L/kg), whilst other authors reported much lower values [2,16,20,27]. 

The Cl_t_ with mean values >10 L/h (a little higher than the glomerular filtration rate (9 L/h)) and the Cl_r_ (approximately 5 L/h) were not far from those previously reported by Segre et al. [16] (8.3 and 7 L/h, respectively), Wenzler et al. [21] (20.4–22.2 L/h and 7.1–8.1 L/h, respectively) and Goto et al. [2,27] (Cl_t_ = 9.0 L/h), whilst Borsa et al. [2,26] reported higher values for both parameters. 

The inconsistencies between the present study results and some data published in the literature, particularly in the old studies, might have several possible explanations. The fosfomycin concentrations were generally assayed in serum instead of plasma and applying less sensitive bioanalytical methods, e.g., microbiological assays or gaschromatographic methods. Another possible explanation could be that blood sampling was interrupted early (12 or 24 h postdose), thus preventing the elimination curve construction up to levels returning below the quantification limit. Furthermore, the methodological differences in the way the pharmacokinetic parameters were calculated and inadequate study sample sizes could explain some of the above-discussed inconsistencies. 

Fosfomycin is water-soluble and has low protein binding as shown in a pooled human serum using ultrafiltration at 37 °C and at a concentration of 20 µg/mL [28]. Thanks to its peculiar chemical nature, fosfomycin is endowed with high tissue dissemination, as also indicated by the pharmacokinetic parameters analysed in the present study. In addition to the blood stream, several literature studies found conspicuous fosfomycin levels in kidneys, bladder, prostate, lungs, bone and cerebrospinal fluid, as well as in inflamed tissues and abscess fluid [2,29,30,31,32,33,34,35,36,37,38,39].

In particular, Borghi et al. [38] measured fosfomycin concentrations in serum and prostatic tissue in 12 healthy men undergoing transurethral resection of the prostate for prostatic adenoma. Six of the twelve patients took a single 3 g dose of fosfomycin trometamol 3 h and the other six, 12 h before surgery. In the former, the fosfomycin in the prostate was on average 20.78 ± 2.35 μg/g concomitantly with serum fosfomycin concentrations of 25.63 ± 3.66 µg/mL, and in the latter, the prostatic fosfomycin was 4.92 ± 0.89 µg/g with a concomitant serum concentration of 6.75 ± 0.82 µg/mL. More recently, Gardiner et al. [34] measured fosfomycin concentrations in serum, urine and prostatic tissue, where the transition zone and peripheral zone were distinguished, in healthy men undergoing a transurethral resection of the prostate for benign prostatic hyperplasia, thus confirming the results of Borghi. After a single 3 g dose of fosfomycin trometamol administered within 17 h before surgery, fosfomycin in the prostate was on average 6.5 ± 4.9 μg/g (range, 0.7–22.1 μg/g), with therapeutic plasma concentrations detectable up to 17 h following the dose.

The healthy male volunteers recruited in the present study performed the collection of the semen from a single ejaculation as multiple fractions. Fosfomycin was quantifiable in the majority of ejaculate fractions from the majority of subjects, both on Day 1 and Day 4. Interestingly, the fosfomycin levels in the seminal plasma through the five collected ejaculate fractions showed a concentration gradient similar to that of PSA, i.e., decreasing in value from fraction 1 to 5, thus indicating a higher concentration of the antibiotic in the prostate than in the seminal vesicles, similar to PSA, which is biologically secreted by the prostate and not by seminal vesicles. The fosfomycin concentration in the seminal plasma slightly increased on average after two consecutive doses compared to the single dose, which was consistent with the results obtained from the plasma. 

*C_P_* and *C_V_* clearly showed that the antibiotic concentration in both the prostate and the seminal vesicles increased on average after two consecutive doses compared to single dosing. Remarkably, *C_P_* on Day 4 was almost four-fold higher than on Day 1, and *C_V_* was approximately seven times as high as the Day 1 estimate. These notable increments in tissue concentrations did not parallel the meaningless increases observed in plasma and highlighted the effect exerted by the second fosfomycin trometamol dose on the fosfomycin concentrations in the tissues. This result demonstrated that fosfomycin is able to distribute into the prostate and into seminal vesicles after one single fosfomycin trometamol dose, which is consistent with the data published by Borghi [38] and Gardiner [34] and that a second consecutive dose increases the antibiotic availability inside these peripheral tissues.

## 5. Conclusions

In conclusion, the study results support the proposed two-dose regimen for fosfomycin indication in perioperative antibiotic prophylaxis for transrectal prostate biopsy. The presented pharmacokinetics data provide further support for the need of a second dose of fosfomycin trometamol about 27 h after the almost complete elimination of the antibiotic after the first dose. The second dose allows fosfomycin to redistribute into the target tissues and, on the basis of the hypothesis that the antibiotic thus, consequently, reattains effective concentrations at the site of action, the second dose could allow the eradication of pathogen foci remnant after the first dose. Indeed, the plasma fosfomycin pharmacokinetic profile observed in the present study showed that the plasma levels of the antibiotic decreased after the first dose and were on average 1.073 ± 0.558 µg/mL at predose before the second of the two administrations. The study results show that the second dose was necessary to boost the concentrations of the antibiotic again both in the central and peripheral compartments, which thus could allegedly reattain effective levels at the target site of action. This study provides further pharmacological support to the fosfomycin trometamol prophylactic indication which was explicitly included among antibiotic prophylaxes recommended by the European Association of Urology in the case of transrectal prostate biopsy [11].

## Figures and Tables

**Table 1:** Mean (±SD) baseline demographic data (N = 24).

Demographic Data	N = 24
**Age (years)**	
Mean ± SD	49.8 ± 6.5
Median (range)	48.0 (40–63)
**Body weight (kg)**	
Mean ± SD	75.20 ± 8.83
Range	73.80 (60.7–92.5)
**Height (cm)**	
Mean ± SD	176.0 ± 5.5
Median (range)	176.0 (165–187)
**BMI**	
Mean ± SD	24.33 ± 2.89
Median (range)	23.85 (19.0–29.6)
**Race**	
White—n (%)	22 (91.7%)
Black—n (%)	1 (4.2%)
Mestizo—n (%)	1 (4.2%)

SD: standard deviation; BMI: body mass index.

**Table 2:** Pharmacokinetic parameters of plasma fosfomycin measured and calculated on Days 1 and 4 after one and two consecutive doses of fosfomycin trometamol to healthy subjects (N = 24).

Parameter (Unit)	Day 1	Day 4	Parameter (Unit)	Day 4
C_max_ (µg/mL)	35.30 ± 7.86	38.98 ± 9.40	C_max,ss_ (µg/mL)	38.98 ± 9.40
t_max_ (h)	2.5 (1.5–4.0)	2.0 (1.5–3.5)	t_max,ss_ (h)	2.0 (1.5–3.5)
AUC_0–t_ (µg/mL∙h)	251.44 ± 45.84	277.06 ± 64.91	AUC_0-t,ss_ (µg/mL∙h)	277.10 ± 64.87
AUC_0–12_ (µg/mL∙h)	203.70 ± 36.15	223.42 ± 48.57	AUC_τ,ss_ (µg/mL∙h)	270.32 ± 60.16
AUC_0–24_ (µg/mL∙h)	242.11 ± 42.93	265.74 ± 58.51	C_ave,ss_ (µg/mL)	10.01 ± 2.23
AUC_0–__∞_ (µg/mL∙h)	261.53 ± 46.72	289.34 ± 72.82	C_min,ss_ (µg/mL)	1.14 ± 0.53
t_1/2_ (h)	8.25 ± 4.29	7.72 ± 3.49	PTF (%)	379.32 ± 56.11
V_z_ (L)	139.72 ± 71.43	118.23 ± 52.68		
Cl_t_ (L/h)	11.85 ± 2.28	10.95 ± 2.55		

Mean ± SD except for t_max_ for which median (range) is shown; SD: standard deviation; AUC_0–t_: area under the concentration–time curve from time zero to time t; AUC_0–__∞_: area under the concentration vs. time curve up to infinity; AUC_0–t,ss_: area under the concentration–time curve at steady-state; AUC_τ,ss_: area under the concentration–time curve at steady-state in the τ interval; AUC_0–12_: area under the concentration–time curve from dose to 12 h postdose; AUC_0–24_: area under the concentration–time curve from dose to 24 h postdose; Cl_t_: total body clearance; Cl_r_: renal clearance; C_max_: peak drug concentration; C_max,ss_: maximum plasma concentration at steady-state; C_min,ss_: minimum plasma concentration at steady-state; C_ave,ss_: average plasma concentration at steady-state; PTF%: peak-trough fluctuation; t_1/2_: half-life; t_max_: time to achieve C_max_; t_max,ss_: time to achieve C_max,ss_; and V_z_: volume of distribution associated with the terminal slope following extravascular administration.

**Table 3:** Total amount of fosfomycin excreted in urine and renal clearance (Cl_r_) calculated on Days 1 and 4 after one and two consecutive doses of fosfomycin trometamol (N = 24).

Variable (Unit)	Day 1	Day 4
Ae_0–t_ (mg)	1270.43 ± 263.09	1397.61 ± 368.21
Fe_0–t_ (%)	42.35 ± 8.77	46.59 ± 12.27
Cl_r_ (L/h)	4.92 ± 1.00	4.87 ± 0.86

Mean ± SD is reported; SD: standard deviation; Ae_0–t_: total amount of fosfomycin excreted in urine from dosing up to 36 h; Fe_0–t_: total fraction of fosfomycin dose excreted in urine from dosing up to 36 h; and Cl_r_: renal clearance.

**Table 4:** Mean (±SD) fosfomycin concentrations (µg/mL) measured in seminal plasma on Days 1 and 4 after one and two consecutive doses of fosfomycin trometamol (N = 22).

Ejaculate Fraction	N	Day 1	N	Day 4
1	18	6.6 ± 7.8	20	25.2 ± 42.8
2	19	4.8 ± 5.5	22	16.9 ± 25.5
3	18	3.0 ± 2.8	19	8.8 ± 8.1
4	16	2.5 ± 2.6	15	13.4 ± 24.3
5	12	1.7 ± 1.9	15	8.7 ± 13.2

Mean ± SD is reported; SD: standard deviation.

**Table 5:** Estimated fosfomycin concentrations in prostate (*C_P_*) and seminal vesicles (*C_V_*) on Days 1 and 4 after single and multiple dose administration of IMP (N = 22).

Variable (Unit)	*C_P_* (µg/mL)		*C_V_* (µg/mL)	
**Day 1**	**9.98**	5.24–14.72	2.08	0.71–3.45
**Day 4**	34.42	18.42–50.43	14.95	0.64–29.27

Mean and 95% confidence interval are reported; *C_p_*: concentration of fosfomycin in prostate; and *C_v_*: concentration of fosfomycin in seminal vesicles.

**Table 6:** Number of subjects reporting and number of reported treatment emergent adverse events by system organ class (SOC) and preferred term (PT) (safety set).

MedDRA Description	Overall (N = 24)	
**SOC**	**AEs** **n**	**Subjects** **n (%)**
PT		
**TEAEs**	**14**	**5 (20.8)**
**Gastrointestinal disorders**	**13**	**5 (20.8)**
Diarrhoea	12	5 (20.8)
Dyspepsia	1	1 (4.2)
**Musculoskeletal and connective tissue disorders**	**1**	**1 (4.2)**
Neck pain	1	1 (4.2)

TEAE: treatment emergent adverse event; AE: adverse event; n: number of adverse events; and n (%): number and percentage of subjects.

## Data Availability

The datasets generated and analysed during the current study are not publicly available. These data are protected by a confidentiality agreement with the study sponsor, Zambon S.p.A., Italy due to their ethically and commercially sensitive nature. Further information about the data and conditions for access are available at www.zambon.com.

## References

[1] Hendlin D., Stapley E.O., Jackson M., Wallick H., Miller A.K., Wolf F.J., Miller T.W., Chaiet L., Kahan F.M., Foltz E.L. (1969). Phosphonomycin, a new antibiotic produced by strains of Streptomyces. Science.

[2] Dijkmans A.C., Ortiz Zacarías N.V., Burggraaf J., Mouton J.W., Wilms E.B., Van Nieuwkoop C., Touw D.J., Stevens J., Kamerling I.M.C. (2017). Fosfomycin: Pharmacological, clinical and future perspectives. Antibiotics.

[3] Popovic M., Steinort D., Pillai S., Joukhadar C. (2010). Fosfomycin: An old, new friend?. Eur. J. Clin. Microbiol. Infect. Dis..

[4] Falagas M.E., Vouloumanou E.K., Samonis G., Vardakas K.Z. (2016). Fosfomycin. Clin. Microbiol. Rev..

[5] Tutone M., Johansen T.E.B., Cai T., Mushtaq S., Livermore D.M. (2022). Susceptibility and Resistance to Fosfomycin and other antimicrobial agents among pathogens causing lower urinary tract infections: Findings of the SURF study. Int. J. Antimicrob. Agents.

[6] Sahu C., Singh S., Patel S.S., Yaduvanshi N., Singh S., Ghoshal U. (2022). Susceptibility profile and clinical response of fosfomycin and other antibiotics against multidrug resistant gram negative urinary isolates: A cross-sectional study. JCDR.

[7] Candel F.J., Matesanz David M., Barberan Lopez J. (2019). New perspectives for reassessing fosfomycin: Applicability in current clinical practice. Rev. Esp. Quimioter..

[8] Freitas D., de Oliveira M., Moreira D.M. (2019). Fosfomycin trometamol vs ciprofloxacin for antibiotic prophylaxis before transrectal ultrasonography-guided prostate biopsy: A meta-analysis of clinical studies. Arab. J. Urol..

[9] Kandil H., Cramp E., Vaghela T. (2016). Trends in antibiotic resistance in urologic practice. Eur. Urol. Focus.

[10] Mazzei T., Diacciati S. (2014). Pharmacological aspects of the antibiotics used for urological diagnostics procedures. J. Chemothr..

[11] Bonkat G., Bartoletti R., Bruyère F., Cal T., Geerlings S.E., Köves B. (2018) EAU Guidelines on Urological Infections. Edn. presented at the EAU Annual Congress Amsterdam the Netherlands 2020. Urological Infections. http://uroweb.org/guidelines/compilations-of-all-guidelines/.

[12] Lundquist F. (1949). Aspects of the biochemistry of human semen. Acta Physiol. Scand..

[13] Cao Y.J., Caffo B., Choi L., Radebaugh C.L., Fuchs E.J., Hendrix C.W. (2008). Noninvasive quantitation of drug concentration in prostate and seminal vesicles: Improvement and validation with desipramine and aspirin. J. Clin. Pharmacol..

[14] Ndovi T.T., Parsons T., Choi L., Caffo B., Rohde C., Hendrix C.W. (2006). A new method to estimate quantitatively seminal vesicle and prostate gland contributions to ejaculate. Br. J. Clin. Pharmacol..

[15] Ndovi T.T., Choi L., Caffo B., Parsons T., Baker S., Zhao M., Rohde C., Hendrix C.W. (2006). Quantitative assessment of seminal vesicle and prostate drug concentrations by use of a noninvasive method. Clin. Pharmacol. Ther..

[16] Segre G., Bianchi E., Cataldi A., Zannini G. (1986). Pharmacokinetic profile of fosfomycin trometamol (Monuril). Eur. Urol..

[17] Ferrari V., Bonanomi L., Borgia M., Lodola E., Marca G. (1981). A new fosfomycin derivative with much improved bioavailability by oral route. Chemioter. Antimicrob..

[18] Wilson P., Williams J.D., Rolandi E. (1988). Comparative pharmacokinetics of fosfomycin trometamol, sodium fosfomycin and calcium fosfomycin in humans. New Trends in Urinary Tract Infections.

[19] Monuril [Summary of Product Characteristics]. Switzerland: Zambon Schweiz AG, May 2015. https://compendium.ch.

[20] Kirby W.M. (1977). Pharmacokinetics of fosfomycin. Chemotherapy.

[21] Wenzler E., Bleasdale S.C., Sikka M., Bunnell K.L., Finnemeyer M., Rosenkranz S.L., Danziger L., Rodvold K.A., Antibacterial Resistance Leadership Group (2018). Phase I study to evaluate the pharmacokinetics, safety, and tolerability of two dosing regimens of oral fosfomycin tromethamine in healthy adult participants. Antimicrob. Agents Chemother..

[22] Bergan T., Thorsteinsson S.B., Albini E. (1993). Pharmacokinetic profile of fosfomycin trometamol. Chemotherapy.

[23] Bergan T. (1990). Degree of absorption, pharmacokinetics of fosfomycin trometamol and duration of urinary antibacterial activity. Infection.

[24] Bergan T., Mastropaolo G., Di Mario F., Naccarato R. (1988). Pharmacokinetics of fosfomycin and influence of cimetidine and metoclopramide on the bioavailability of fosfomycin trometamol. New Trends in Urinary Tract Infections.

[25] Muller-Serieys C., Bergogne-Berezin E., Joly-Guillou M.L. (1987). Fosfomycin-trometamol (monuril): Pharmacokinetics and food-drug interactions. Pathol. -Biol..

[26] Borsa F., Leroy A., Fillastre J.P., Godin M., Moulin B. (1988). Comparative pharmacokinetics of tromethamine fosfomycin and calcium fosfomycin in young and elderly adults. Antimicrob. Agents Chemother..

[27] Goto M.I.T.S., Sugiyama M.A.S.A., Nakajima S.H.I.N., Yamashina H.A.J.I.M.E. (1981). Fosfomycin kinetics after intravenous and oral administration to human volunteers. Antimicrob. Agents Chemother..

[28] Kestle D.G., Kirby W.M.N. (1969). Clinical pharmacology and in vitro activity of phosphonomycin. Antimicrob. Agents Chemother.

[29] Pfausler B., Spiss H., Dittrich P., Zeitlinger M., Schmutzhard E., Joukhadar C. (2004). Concentrations of fosfomycin in the cerebrospinal fluid of neurointensive care patients with ventriculostomy-associated ventriculitis. J. Antimicrob. Chemother..

[30] Frossard M., Joukhadar C., Erovic B.M., Dittrich P., Mrass P.E., Van Houte M., Burgmann H., Georgopoulos A., Müller M. (2000). Distribution and antimicrobial activity of fosfomycin in the interstitial fluid of human soft tissues. Antimicrob. Agents Chemother..

[31] Matzi V., Lindenmann J., Porubsky C., Kugler S.A., Maier A., Dittrich P., Smolle-Jüttner F.M., Joukhadar C. (2010). Extracellular concentrations of fosfomycin in lung tissue of septic patients. J. Antimicrob. Chemother..

[32] Joukhadar C., Klein N., Dittrich P., Zeitlinger M., Geppert A., Skhirtladze K., Frossard M., Heinz G., Müller M. (2003). Target site penetration of fosfomycin in critically ill patients. J. Antimicrob. Chemother..

[33] Sauermann R., Karch R., Langenberger H., Kettenbach J., Mayer-Helm B., Petsch M., Wagner C., Sautner T., Gattringer R., Karanikas G. (2005). Antibiotic abscess penetration: Fosfomycin levels measured in pus and simulated concentration-time profiles. Antimicrob. Agents Chemother..

[34] Gardiner B.J., Mahony A.A., Ellis A.G., Lawrentschuk N., Bolton D.M., Zeglinski P.T., Frauman A.G., Grayson M.L. (2014). Is fosfomycin a potential treatment alternative for multidrug-resistant gram-negative prostatitis?. Clin. Infect. Dis..

[35] Müller O., Rückert P.D.D., Walter W., Haag R., Sauer W. (1982). Fosfomycin-Konzentrationen im Serum und in der Galle. Infection.

[36] Schintler M.V., Traunmüller F., Metzler J., Kreuzwirt G., Spendel S., Mauric O., Popovic M., Scharnagl E., Joukhadar C. (2009). High fosfomycin concentrations in bone and peripheral soft tissue in diabetic patients presenting with bacterial foot infection. J. Antimicrob. Chemother..

[37] Legat F.J., Maier A., Dittrich P., Zenahlik P., Kern T., Nuhsbaumer S., Frossard M., Salmhofer W., Kerl H., Muller M. (2003). Penetration of fosfomycin into inflammatory lesions in patients with cellulitis or diabetic foot syndrome. Antimicrob. Agents Chemother..

[38] Borghi C.M., La Veneziana D., Riva A., Marca G., Zanninin G. (1986). Concentrazioni nel siero e nel tessuto prostatico di fosfomicina con fosfomicina-trometamolo, nuovo derivato della fosfomicina con elevata biodisponibilita per somministrazione orale. Farm. Ter..

[39] Scaglione F., Cicchetti F., Demartini G., Arcidiacono M. (1994). Fosfomycin distribution in the lower urinary tract after administration of fosfomycin trometamol salt. Int. J. Clin. Pharmacol. Res..

